# Vitamin A Modulates AHR Signaling and Restricts Zika Virus Replication in Human Retinal Pigment Epithelial Cells: Insights from Molecular Modeling and Antiviral Assays

**DOI:** 10.3390/pathogens15050518

**Published:** 2026-05-12

**Authors:** Agostina B. Marquez, Priscila A. Lanza Castronuovo, Cecilia L. Barbieri, Mayra A. Castañeda Cataña, Claudia S. Sepúlveda, Agustina Alaimo, D. Mariano A. Vera, Cybele C. García

**Affiliations:** 1 Laboratorio de Estrategias Antivirales, Departamento de Química Biológica, Facultad de Ciencias Exactas y Naturales, Universidad de Buenos Aires (UBA), Intendente Güiraldes 2160, Ciudad Autónoma de Buenos Aires 1428, Argentina; 2 Instituto de Química Biológica de la Facultad de Ciencias Exactas y Naturales (IQUIBICEN), Consejo Nacional de Investigaciones Científicas y Técnicas (CONICET)–UBA, Intendente Güiraldes 2160, Ciudad Autónoma de Buenos Aires 1428, Argentina; 3 QUIAMM-INBIOTEC, Departamento de Química y Bioquímica, Facultad de Ciencias Exactas y Naturales, Universidad Nacional de Mar del Plata, Funes 3350, Mar del Plata 7600, Argentina; 4 Comisión de Investigaciones Científicas (CIC), Calle 526 entre 10 y 11, La Plata 1900, Argentina

**Keywords:** zika virus (ZIKV), vitamin A, retinal pigment epithelium (RPE), host-directed antiviral therapy, molecular dynamics simulations, aryl hydrocarbon receptor (AHR), antiviral combination therapy, resveratrol

## Abstract

Zika virus (ZIKV) is an emerging flavivirus associated with congenital malformations and ocular complications, representing a significant public health concern. Retinal pigment epithelium (RPE) cells play a key role in maintaining retinal integrity and represent a primary target of ZIKV infection, making them a relevant model for studying host–virus interactions. In this study, we evaluated the antiviral activity of fat- and water-soluble vitamins against ZIKV in hTERT RPE-1 (hRPE1) cells. Particularly, vitamin A was identified as the compound that most effectively inhibited viral replication. Molecular dynamics simulations focusing on the PAS-B domain of the aryl hydrocarbon receptor (AHR) revealed a high affinity of vitamin A for the receptor. In hRPE1 cells, vitamin A treatment reduced viral RNA levels and decreased *CYP1A1*, *TDO*, and *AHR* mRNA expression. In parallel, *IFNB1* expression increased, consistent with the involvement of type I interferon (IFN-I), as no antiviral effect was observed in IFN-I-deficient Vero cells. These findings suggest that vitamin A restricts ZIKV replication through host antiviral responses, potentially involving modulation of AHR-associated signaling. The combination of vitamin A and the well-known polyphenol resveratrol further enhanced antiviral activity, showing predominantly additive effects. Together, these results support the potential use of both bioactive compounds as a combined therapeutic strategy.

## 1. Introduction

Zika virus (ZIKV) is a mosquito-borne flavivirus that emerged as a major global health concern following the 2015–2016 outbreaks in the Americas. Although ZIKV infection is often asymptomatic or mild in adults, it has been strongly associated with severe neurological complications, including Guillain–Barré syndrome, as well as congenital abnormalities collectively termed congenital Zika syndrome [1]. In addition to neurodevelopmental defects, accumulating clinical evidence indicates that ZIKV infection can cause significant ocular abnormalities, particularly in newborns exposed in utero. Ocular involvement occurs in up to 70% of affected infants and includes iris coloboma, lens subluxation, cataracts, and congenital glaucoma, although the most characteristic alterations involve the posterior segment of the eye. Histopathological analyses of eyes from deceased fetuses have revealed perivascular inflammatory infiltrates in a markedly thinned choroid, degeneration of the retinal pigment epithelium, and optic nerve atrophy [2,3].

Retinal pigment epithelial (RPE) cells form a postmitotic monolayer located between the neural retina and the choroid and play a central role in retinal homeostasis, including maintenance of the blood–retina barrier (BRB), regulation of photoreceptor function, and modulation of innate immune responses [4]. In ocular tissues, RPE cells have been identified as a primary target of ZIKV infection. Currently, no specific antiviral therapy is available for ZIKV infection, and clinical management remains largely supportive. In cases with ocular involvement, treatment focuses on managing specific complications or visual impairment. Disruption of the RPE can provide an entry route for viruses that cross the fenestrated choroidal capillaries to infiltrate neural retinal tissue. Nevertheless, the precise mechanisms behind the development of ocular complications and the fragilization of the BRB following ZIKV infection remain unclear [5]. Moreover, there are currently no clinical guidelines or trials for the treatment of ZIKV-associated ocular disease, and management remains largely supportive and symptom-based [6]. Consequently, RPE cells constitute a physiologically relevant model for studying host-directed antiviral strategies in the context of ZIKV-associated ocular disease.

Host-targeted antiviral approaches have gained increasing attention as complementary strategies to direct-acting antivirals. By modulating cellular pathways required for viral replication, host-directed therapies may reduce the likelihood of viral resistance and influence infection outcomes through immune regulation [7]. Several studies have highlighted the promising biological properties of plant-derived bioactive compounds, including proteins, polyphenols, and vitamins, which promote both animal and human health. These molecules have emerged as attractive candidates due to their structural diversity, safety profiles, relative abundance, cost-effectiveness, and ability to modulate cellular signaling pathways involved in immunity, metabolism, and cellular stress responses [8].

Vitamins are essential micronutrients required for normal physiological function, and deficiencies in these bioactives have been associated with increased susceptibility to viral infections and impaired immune responses [9]. Beyond their classical nutritional roles, several vitamins have been shown to regulate intracellular signaling pathways, oxidative stress responses, and innate immune activation. Although vitamins have been associated with antiviral and immunomodulatory effects, to the best of our knowledge, no studies have investigated their antiviral activity against ZIKV infection in ocular tissues, particularly in retinal cells.

Among host pathways implicated in ZIKV infection, the aryl hydrocarbon receptor (AHR) has emerged as a critical proviral factor. AHR is a ligand-activated transcription factor belonging to the basic helix–loop–helix/Per-Arnt-Sim (bHLH-PAS) family. In its inactive state, AHR resides in the cytoplasm as part of a multiprotein chaperone complex. Upon ligand binding to its PAS-B domain, the receptor translocates to the nuclear compartment, where it regulates the transcription of genes involved in xenobiotic metabolism and immune modulation [10]. Our previous studies have demonstrated that AHR activation during ZIKV infection suppresses type I interferon (IFN-I) responses, thereby promoting viral replication [11,12]. Mechanistically, AHR activation induces the expression of indoleamine-2,3-dioxygenase (IDO) and tryptophan-2,3-dioxygenase (TDO), by increasing the production of kynurenine, an endogenous ligand that further activates AHR through a positive feedback loop.

Pharmacological inhibition of AHR using the selective antagonist CH-223191 significantly reduces ZIKV replication in vitro and in vivo, indicating AHR signaling as a potential therapeutic target during infection [11].

Importantly, AHR signaling operates in RPE cells and contributes to cellular adaptation to environmental stress and immune regulation [13,14]. Structural and computational studies have identified the PAS-B domain as the principal ligand-binding region of AHR and the primary target of both agonists and antagonists, making it a suitable focus for molecular docking and simulation studies [15].

Based on the above-mentioned evidence, we hypothesized that vitamin A, which regulates gene transcription and immune responses in ocular tissues, may modulate AHR signaling during ZIKV infection [16]. To test this hypothesis, we first performed an antiviral screening of multiple vitamins in ZIKV-infected hRPE cells and subsequently conducted mechanistic analyses focused on vitamin A. These studies included combination treatments, molecular dynamics (MD) simulations to explore potential interactions between vitamin A and the AHR PAS-B domain, and analysis of AHR-dependent gene expression to determine pathway modulation.

## 2. Results

### 2.1. Effects of Vitamin Treatments on ZIKV Replication in hRPE1 Cells

**Figure 1:**
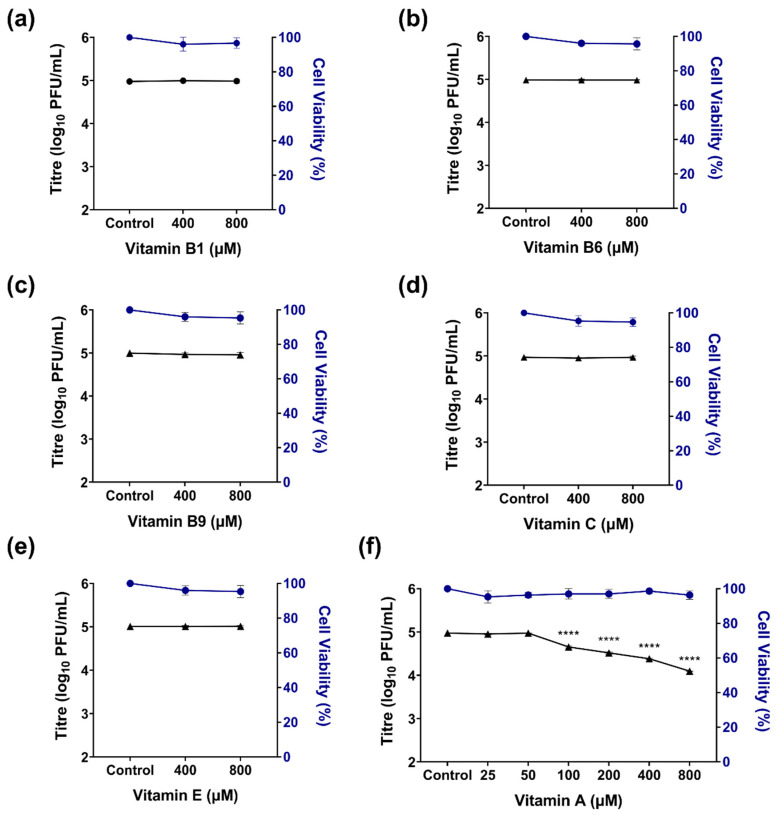
Antiviral activity and cell viability of vitamins in ZIKV-infected hRPE1 cells. Cells were infected with ZIKV (MOI = 0.1) and treated with increasing concentrations of (**a**) vitamin B1, (**b**) vitamin B6, (**c**) vitamin B9, (**d**) vitamin C, (**e**) vitamin E, and (**f**) vitamin A. Antiviral activity (black lines) was assessed at 48 h post-infection by quantification of infectious viral particles in the supernatant using a plaque assay. Cell viability (blue lines) was determined at 72 h using the crystal violet assay. Data are expressed as mean ± SD (*n* = 3 independent experiments). **** *p* < 0.0001 compared to untreated control.

To evaluate the antiviral activity of different vitamins against ZIKV, hTERT-RPE1 (hRPE1) cells were first subjected to a cytotoxicity assessment. Cells were exposed for 72 h to increasing concentrations (200–800 µM) of vitamin A (retinyl palmitate), vitamin B1, vitamin B6, vitamin B9, vitamin C, and vitamin E, and cell viability was assessed using a crystal violet assay, which estimates the number of adherent cells based on dye retention. None of the tested vitamins exhibited significant cytotoxic effects within the evaluated concentration range, maintaining cell viability above 90% (Figure 1).

Based on these results, antiviral activity was assessed by quantifying viral titers in supernatants from infected hRPE1 cells treated with each vitamin. Initial screening at 400 and 800 µM revealed that vitamin A significantly reduced ZIKV viral yield, whereas vitamins B1, B6, B9, C, and E showed no clear antiviral activity under the tested conditions (Figure 1a–e). Given the pronounced antiviral effect observed with vitamin A, additional experiments were performed using lower concentrations (50–200 µM). A significant decrease in viral titers was detected down to 100 µM, while cell viability remained unaffected (Figure 1f). The calculated EC_50_ value for vitamin A was 208.35 µM. Higher concentrations could potentially further refine the EC_50_ estimation; however, they were not explored due to solubility and physiological relevance constraints.

### 2.2. Combination Therapy of Vitamin A and Resveratrol

In a previous study, we demonstrated that resveratrol (RES) inhibits ZIKV replication in this cell line with a moderate antiviral effect [17]. Based on these findings and considering that combination strategies can enhance antiviral efficacy while allowing dose reduction, we evaluated the effect of vitamin A and RES co-treatment in hRPE1 cells. Vitamin A exhibited a moderate but significant antiviral effect on ZIKV replication, although high concentrations were required to achieve this effect. Therefore, we assessed a range of concentrations of vitamin A (25–800 μM) and RES (3–50 μM) in combination.

**Figure 2:**
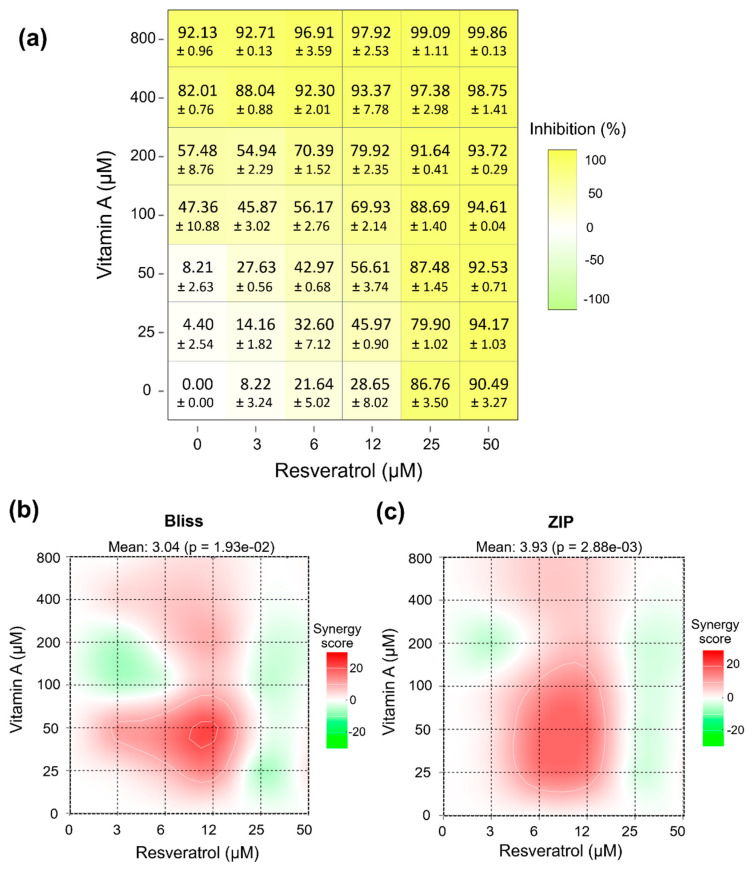
Dose–response matrix and drug interaction analysis of vitamin A and RES. (**a**) Percentage of inhibition across the full concentration matrix of vitamin A (25–800 µM) and RES (3–50 µM). Values represent mean inhibition percentages for each drug combination. (**b**) Synergy landscape calculated using the Bliss Independence reference model. The color scale represents synergy scores, where positive values (red) indicate synergistic interactions, negative values (green) indicate antagonism, and values around zero correspond to additive effects. The mean Bliss synergy score and associated *p*-value are shown above the plot. (**c**) Synergy landscape calculated using the ZIP model. Color coding and score interpretation are as described for panel (**b**). The mean ZIP synergy score and *p*-value are indicated above the plot.

First, cytotoxicity of the combined treatments was evaluated within the tested ranges (25–800 μM for vitamin A and 3–50 μM for RES). No significant cytotoxicity was observed under these conditions; therefore, these concentrations were used for antiviral assays. The inhibition matrix obtained for the combination of vitamin A (0–800 µM) and RES (0–50 µM) showed a clear concentration-dependent effect for both compounds (Figure 2a). RES alone (0 µM vitamin A) induced a progressive increase in inhibition, rising from 8.22% to 89.3% as the concentration was raised from 3 to 0 µM. Similarly, vitamin A alone (0µM RES) exhibited a dose-dependent effect, with inhibition increasing from 4.4% to over 90% at the highest concentration tested (800 µM). When combined, the inhibitory effect increased further as both concentrations rose. At intermediate concentration ranges (e.g., 100–200 µM vitamin A combined with 12–25 µM RES), inhibition values already exceeded 70–90%. At the highest concentration combinations (≥400 µM vitamin A and ≥25 µM RES), inhibition approached maximal levels (approximately 97–99.86%). Importantly, at lower concentrations of vitamin A (25–50 µM), where the compound alone produced minimal inhibition, the addition of RES markedly increased the inhibitory effect. These combinations produced substantially higher inhibition than the vitamin A monotherapy at the same concentrations, indicating an enhanced effect of the combined treatment.

To characterize the interaction between vitamin A and RES, combination effects were evaluated using the Bliss Independence and Zero Interaction Potency (ZIP) models implemented in SynergyFinder. Bliss was selected as a reference model to quantify deviations from an expected non-interaction scenario based on probabilistic independence, without requiring prior assumptions regarding the precise molecular mechanisms of each compound [18]. The ZIP model was included as a complementary approach, as it integrates both potency shifts and response surface deviations to assess interaction effects across the full dose–response matrix [19,20]. According to the Bliss model (Figure 2b), the mean synergy score (δ-score) was 3.04 (*p* = 1.93 × 10^−2^), which falls within the range generally considered additive (−10 to +10). Similarly, ZIP analysis (Figure 2c) yielded a mean score of 3.93 (*p* = 2.88 × 10^−3^), also consistent with an overall additive interaction. Despite this global additive profile, localized regions of the dose–response matrix exhibited positive synergy scores (red areas), particularly at intermediate concentrations of both compounds. Conversely, limited areas with negative scores (green regions) suggested mild antagonistic deviations at specific concentration combinations. At higher concentration ranges, interaction scores tended to approach zero, consistent with response saturation near maximal inhibition.

Overall, both models consistently indicate that the interaction between vitamin A and RES is globally additive.

### 2.3. In Silico Analysis of Vitamin A and AHR Interactions

To gain mechanistic insight into the antiviral activity of vitamin A, we investigated its potential interaction with the ligand-binding domain of the AHR. Vitamin A comprises a group of retinoids, including retinol and retinyl esters such as retinyl palmitate [21]. Given that retinyl palmitate is known to undergo intracellular hydrolysis after uptake, in silico analyses were conducted using all-trans-retinol, a biologically relevant vitamin A metabolite suitable for evaluating potential interactions with intracellular targets [22]. RPE cells have been reported to possess retinyl ester hydrolase activity capable of converting retinyl esters into retinol [23,24,25]. Since the PAS-B domain constitutes the primary ligand-binding pocket of AHR, a computational workflow combining molecular docking and MD simulations was employed to evaluate binding pose, stability, and interaction patterns.

The docking protocol was first validated by re-docking indirubin, the co-crystallized ligand of the AHR PAS-B domain, and the predicted pose was very close to the experimental structure as shown in Figure S2. CH-223191 and TCDD were used as reference AHR antagonist and agonist ligands, respectively, while vitamin B6 was included as a negative control. Vitamin B6 was included as a negative control based on its lack of antiviral activity in our experimental screening, allowing comparison with a compound not expected to interact with the AHR binding pocket.

Molecular Mechanics Generalized Born Surface Area (MMGBSA) binding free energy calculations indicated that vitamin A exhibits a favorable predicted interaction with the AHR ligand-binding domain, with values comparable to those obtained for reference ligands, stronger than the crystallographic indirubin and largely more favored than that obtained for vitamin B6 (Table 1). While these results do not completely demonstrate functional antagonism, they strongly suggest that all-trans-retinol is compatible with the AHR binding pocket and may influence receptor activity.

**Figure 3:**
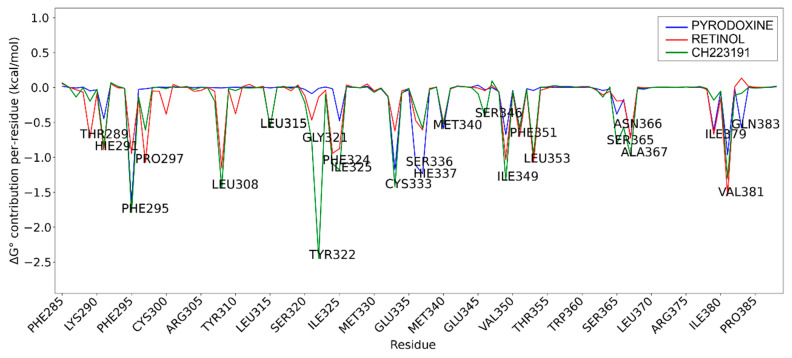
MD analysis of residue-specific contribution of binding free energies for pyridoxine, all-trans-retinol, and the AHR antagonist CH-223191.

Per-residue decomposition of the binding free energy obtained from MD simulations was performed for pyridoxine (vitamin B6), all-trans-retinol, and the selective AHR antagonist CH-223191 in the PAS-B domain of AHR. Residues displaying the most pronounced negative energy contributions indicate key stabilizing interactions within the ligand-binding pocket. This comparative analysis highlights differences in binding patterns and interaction profiles among the three compounds (Figure 3).

The per-residue decomposition of binding free energies revealed a high degree of similarity between all-trans-retinol and the selective AHR antagonist CH-223191, whereas pyridoxine exhibited a markedly distinct and weaker interaction profile, supporting its role as a negative control.

**Figure 4:**
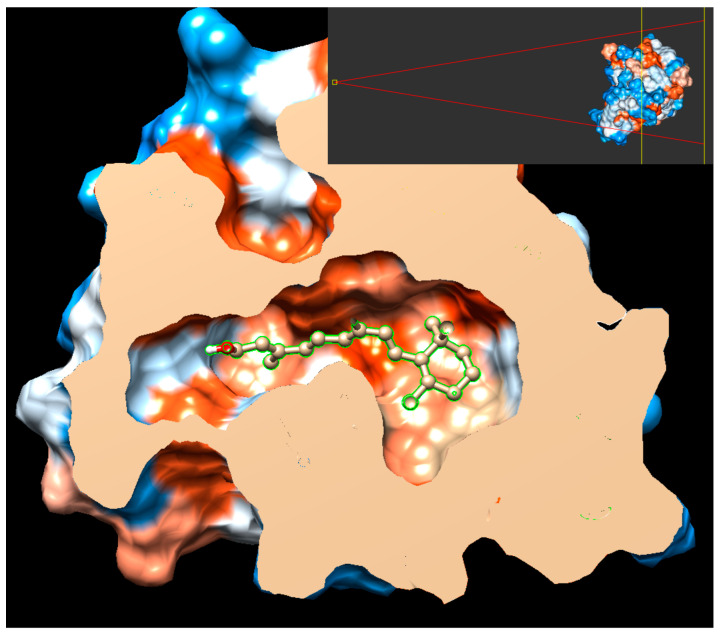
Cortex of the AHR-PAS-B domain represented as a hydrophobicity molecular surface (orange = maximum hydrophobicity) showing the pose of a representative structure of the most visited cluster after a cluster analysis of the trajectory during the last 90 ns of simulation. The upper right inset shows the plane of the cortex. Further details in Figure S3.

Vitamin A and CH-223191 showed strong stabilizing interactions at several key residues within the PAS-B binding pocket, including PHE295, HIS291, LEU308, LEU353, and SER366, indicating a comparable engagement of critical structural elements involved in ligand recognition and receptor modulation. Notably, both compounds exhibited pronounced negative energy contributions at hydrophobic residues lining the core of the binding cavity, consistent with a stable binding mode and effective pocket occupancy, as shown in Figure 4. This behavior was maintained throughout the simulation (see also Supporting Information, Figure S3).

In contrast, pyridoxine showed substantially reduced stabilization across the majority of these residues, characterized by weaker energy contributions and a predominance of transient polar interactions. The absence of significant stabilization at central hydrophobic residues suggests a limited capacity of pyridoxine to occupy and stabilize the PAS-B binding pocket, consistent with a low-affinity binding mode.

Subtle differences between all-trans-retinol and CH-223191 were observed, including enhanced stabilization of vitamin A at VAL381 and stronger contributions of CH-223191 at TYR322 and CYS333. Nevertheless, the overall interaction patterns remain highly comparable, supporting a similar binding orientation and mode of receptor engagement.

Collectively, these results indicate that all-trans-retinol mimics the binding behavior of the canonical AHR antagonist CH-223191, whereas pyridoxine lacks the structural and energetic features required for stable engagement of the PAS-B domain, which may account for its less favorable estimated binding free energy in the MD simulations.

**Figure 5:**
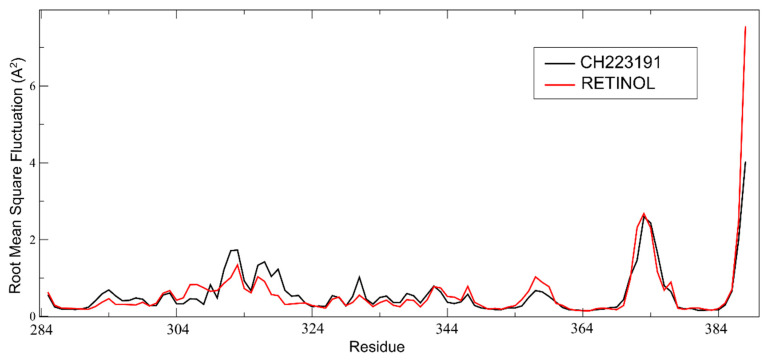
Comparison of the RMSF per-residue of the AHR PAS-B domain calculated over the final 50 ns of the MD simulation for reference compound CH223191 and all-trans-retinol.

In the analysis of the AHR PAS-B domain’s root mean square fluctuation (RMSF) per residue over the last 50 ns of MD simulations with all-trans-retinol and CH223191, we observed that specific regions display similar flexibility profiles in both ligand-bound states. Notably, the central antiparallel β-sheet core of the PAS-B domain exhibited consistently low fluctuations, indicating preserved structural stability regardless of the bound ligand. This observation is consistent with the structural organization of the PAS-B domain, which adopts a canonical PAS fold consisting of a β-sheet scaffold flanked by α-helices [15]. Furthermore, the α-helical segments adjacent to the β-sheet, particularly helices αC and αD, showed comparable fluctuation patterns in both simulations, suggesting that these elements contribute similarly to the conformational dynamics of the PAS-B domain in each ligand-bound condition (Figure 5).

#### 2.3.1. Analysis of mRNA Expression Associated with the AHR Signaling Pathway

To evaluate whether vitamin A is indeed modulating the AHR signaling pathway, the mRNA expression of relevant proteins in this pathway was evaluated. The *AHR*, *TDO* and *CYP1A1* mRNA relative expressions were determined by RT-qPCR. Additionally, *NS5*-ZIKV RNA expression was also measured.

Previous studies have shown that the effect of CH223191 is more pronounced when cells are exposed to the compound prior to infection (pre-treatments). Consequently, cells were exposed to this compound (10 μM) and to vitamin A (800 μM) for 2 h, then infected, and subsequently incubated with drug-free medium for 24 h. Following this period, the relative mRNA expressions associated with the AHR signaling pathway were determined by RT-qPCR.

On the other hand, considering that the antiviral activity assays were conducted by treating cells for 48 h post-infection (post-treatment), transcript expression was quantified under these conditions with concentrations of vitamin A at 400 and 800 μM.

**Figure 6:**
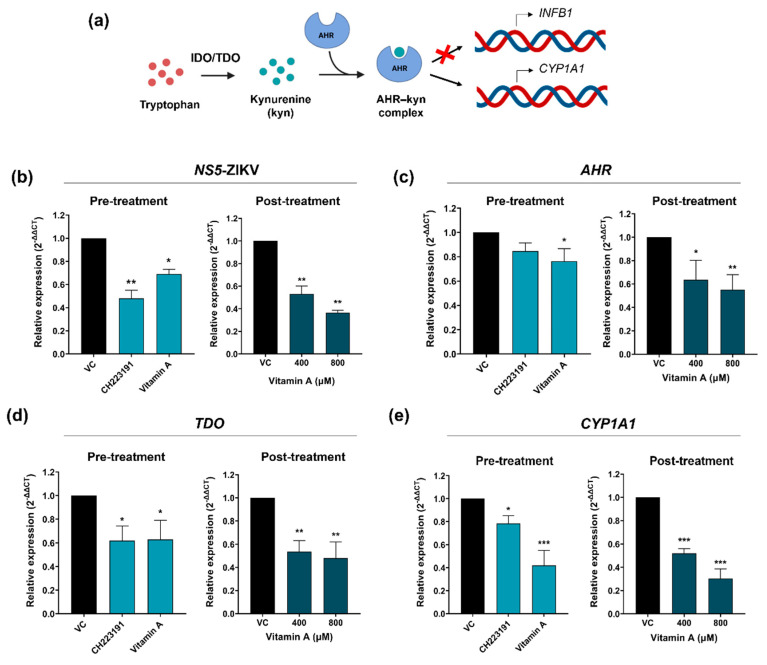
Study of the AHR signaling pathway. (**a**) Schematic illustration of AHR activation (created with BioRender). IDO/TDO-mediated conversion of tryptophan into kynurenine leads to AHR activation, resulting in CYP1A1 induction and IFNB1 repression. The RNA expression of (**b**) *NS5*-ZIKV and mRNA expression of (**c**) *AHR*, (**d**) *TDO* and (**e**) *CYP1A1* were determined by RT-qPCR. For pre-treatments, cells were treated with CH223191 (10 μM) and vitamin A (800 μM) for 2 h and then infected with ZIKV (MOI = 0.1). The inocula were removed and medium without drug was added. Cells were incubated for 24 h at 37 °C. For post-treatments, cells were infected with ZIKV (MOI = 0.1) and then treated for 48 h with 400 and 800 μM concentrations of vitamin A. Total RNA was extracted from cells and viral RNA or cellular mRNA relative expression were determined by qRT-PCR. Expression values are relative to infected untreated cells (viral control, VC). Data are expressed as mean ± SD (*n* = 3 experiments). *** *p*  <  0.001, ** *p* < 0.01, * *p* < 0.05 compared to VC.

A decrease in the expression of *NS5*-ZIKV RNA was observed in pre-treatment conditions, for both the antagonist CH223191 and for vitamin A. Additionally, consistent with the results obtained from the assessment of antiviral activity, a reduction in *NS5*-ZIKV RNA abundance was observed in post-treatment conditions for both 400 and 800 μM concentrations of vitamin A (Figure 6b). Regarding *AHR* mRNA expression, it was found that vitamin A downregulates the expression of this transcript in pre- and post-treatment conditions. Nevertheless, no significant reduction was observed with CH223191 treatments (Figure 6c). Evidence suggests that this antagonist predominantly influences other components in the signaling pathway, and its effect on *AHR* mRNA expression may vary depending on the cell line. Therefore, the expression of the TDO and *CYP1A1* transcripts was quantified. In this case, CH223191 and vitamin A impacted the relative expression levels in both pre- and post-treatment conditions (Figure 6d,e).

Collectively, our findings strongly suggest that vitamin A modulates the AHR signaling pathway in hRPE1 cells.

#### 2.3.2. Assessment of IFNB1 Expression Following Vitamin A Treatment

**Figure 7:**
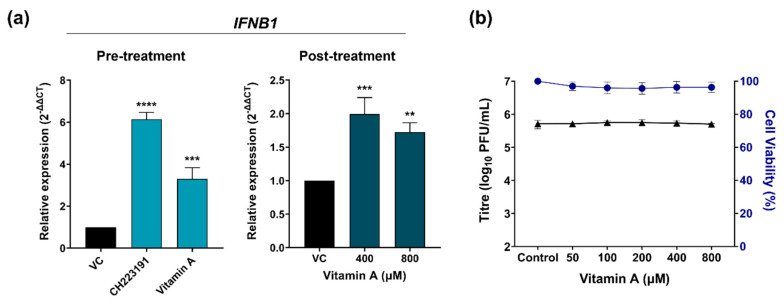
(**a**) *IFNB1* mRNA in hRPE1 cells determined by RT-qPCR. Expression values are relative to untreated infected cells (VC). (**b**) Antiviral activity of vitamin A against ZIKV in Vero cells. The cultures were exposed to ZIKV (MOI = 0.1) for 1 h, after which the inocula were removed. The cells were then treated with vitamin A (50–800 μM) for 48 h, and subsequently, the infectious particles were quantified using a plaque assay. Data are expressed as mean ± SD (*n* = 3 experiments). **** *p*  <  0.0001, *** *p* < 0.001, ** *p* < 0.01, compared to VC.

As mentioned above, AHR antagonists limit AHR activation, enhancing the host antiviral response mediated by IFN-I. For this reason, the expression levels of *IFNB1* were measured in pre-treatments for CH223191 (10 μM) and vitamin A (800 μM) and post-treatments for vitamin A (400 and 800 μM). The results showed that in all cases there was an increase in the relative expression of *IFNB1* mRNA (Figure 7a). On the other hand, to evaluate the relevance of the role of IFN-I in the antiviral effect of vitamin A, the antiviral activity of this compound was evaluated in Vero cells. These cells express AHR but do not produce IFN-I during viral infections due to spontaneous gene deletions. For this, cells were infected with ZIKV (MOI = 0.1) for 1 h; then the inocula were removed and the cells were treated with vitamin A (50–800 μM) for 48 h. Subsequently, the infectious particles were quantified by the plaque assay. The results showed that vitamin A did not inhibit ZIKV in this cell line at any of the concentrations (Figure 7b). All these results together suggest that the antiviral activity of this vitamin is strongly related to the production of IFN-I.

## 3. Discussion

Natural bioactive compounds such as vitamins and polyphenols have attracted increasing attention as potential antiviral agents due to their structural diversity, favorable safety profiles, and ability to modulate host cellular pathways. Many of these molecules regulate processes involved in oxidative stress, inflammation, and innate immune responses, which play critical roles during viral infections [26,27]. In ocular tissues, these pathways are particularly relevant, as RPE cells actively participate in immune regulation and antiviral defense [26]. Therefore, exploring vitamins and polyphenols as modulators of host signaling pathways, including the previously studied AHR [11], may provide a promising strategy to identify host-directed antiviral approaches against ZIKV.

In the present work, the antiviral effects of different vitamins against ZIKV in human RPE cells were evaluated. Among the vitamins tested, vitamin A was identified as the compound producing the strongest reduction in viral replication. Although the active concentrations observed in vitro may exceed those typically achieved under physiological conditions, these experiments were designed to identify potential host-modulatory mechanisms and establish proof-of-concept antiviral activity in a controlled cellular model. These findings are in good agreement with the MD simulations which identified vitamin A as a strong ligand of the AHR-PAS-B. The pose within the hydrophobic pocket closely resembles the one obtained for the known antagonist CH-223191 as demonstrated by the analyses of the interactions and with a comparable free energy of binding.

Combination therapies are widely used in antiviral research to enhance therapeutic efficacy, reduce the required doses of individual compounds, and limit potential toxicity [28]. Based on previous reports demonstrating that RES inhibits ZIKV replication in hRPE cells with moderate efficacy, we selected this compound for combination studies with vitamin A [17]. The combined treatment resulted in a greater reduction in viral titers compared to individual treatments across most of the concentration matrix tested. Synergy analysis indicated that the overall interaction between vitamin A and RES was additive. However, localized regions of modest synergistic and antagonistic interaction were observed at specific intermediate concentrations. This pattern suggests that the interaction between both compounds is not uniform across the dose–response landscape but instead depends on the relative contribution of each compound to the overall antiviral effect. One possible explanation for this behavior lies in the pleiotropic nature of both vitamin A and RES. RES is known to modulate multiple cellular pathways, including redox balance, inflammatory signaling, and host transcriptional responses [29,30,31], while vitamin A regulates gene expression through nuclear receptors and can influence epithelial differentiation and immune-related pathways [32,33,34]. When compounds exert effects through multiple partially overlapping cellular networks, the resulting pharmacodynamic interaction may vary across concentration ranges [35]. At lower or intermediate doses, where neither compound alone achieves maximal pathway engagement, combined modulation of complementary host factors may lead to synergistic suppression of viral replication. In contrast, at higher concentrations where antiviral pathways may already be saturated, the combined effect may shift toward additivity due to a ceiling effect in viral inhibition. Another factor that may contribute to this concentration-dependent interaction is the nonlinear nature of antiviral dose–response relationships. When one compound approaches maximal efficacy, additional pathway modulation by a second compound may produce limited incremental benefit [36]. Conversely, in dynamic regions of the dose–response curve, small cooperative effects can translate into measurable synergistic scores. This may explain why synergy was observed only in defined regions of the matrix rather than across all tested concentrations. Importantly, the additive interaction observed in this study does not diminish the therapeutic relevance of the combination. Additive effects can still allow dose reduction of individual compounds while maintaining antiviral efficacy, which is particularly valuable when high concentrations are required for activity, as observed for vitamin A. Moreover, additive or mildly synergistic combinations are often preferred in antiviral strategies, as they may reduce the risk of viral resistance while maintaining predictable pharmacodynamic behavior. These findings raise the question of which host pathways might mediate the antiviral effect of vitamin A in hRPE cells.

Vitamin A plays a central role in both innate and adaptive immunity, contributing to epithelial integrity, immune cell differentiation, and antiviral cytokine responses [37,38]. Retinoids have been reported to inhibit replication of several viruses in vitro, in part via enhancement of IFN-I signaling [39]. Considering this background and the concentration-dependent interactions of RES, vitamin A may exert antiviral effects through AHR, a receptor linked to innate immunity and IFN-I responses [40]. RES has been shown to engage AHR, but its precise activity, whether as an agonist or antagonist, remains controversial, highlighting the potential for context-dependent effects [41,42,43]. Given the combinatorial data, we proposed AHR as a relevant target for vitamin A and a potential contributor to the observed interactions. Previous results from our laboratory showed that activation of AHR suppresses IFN-I responses through a kynurenine-dependent feedback loop involving enzymes such as TDO and IDO, favoring ZIKV replication, whereas pharmacological inhibition enhances IFN signaling and restricts viral replication [11]. Molecular docking and dynamics simulations indicated that vitamin A can interact with the ligand-binding domain of AHR, providing a structural basis for potential receptor modulation. Consistent with this hypothesis, vitamin A treatment was associated with reduced expression of AHR and its canonical downstream target *CYP1A1*, along with decreased *TDO* expression. In parallel, *IFNB1* mRNA levels increased, whereas no antiviral effect was detected in IFN-deficient Vero cells. These results are compatible with a reduction in AHR pathway activity and partial relief of AHR-mediated suppression of antiviral IFN responses, mirroring transcriptional patterns observed with established AHR antagonists [11]. Together, these findings support a model in which vitamin A functionally attenuates AHR signaling to enhance antiviral IFN responses. However, additional studies, including assessment of AHR nuclear translocation or reporter-based assays, will be required to determine whether vitamin A directly targets AHR.

## 4. Materials and Methods

### 4.1. In Vitro Assays

#### 4.1.1. Chemicals

Vitamin A (Retinyl palmitate), and vitamin E (α-tocopheryl acetate) were kindly provided by Laboratorios Rontag S.A, Provincia de Buenos Aires, Argentina.

Vitamin B1 (thiamine hydrochloride), vitamin B6 (pyridoxine hydrochloride) and vitamin B9 (folic acid) were generously donated by Laboratorios Bagó S.A., Ciudad Autónoma de Buenos Aires, Argentina.

Vitamin C (ascorbic acid) and *trans*-resveratrol were supplied by Laboratorios Temis Lostaló S.A., Argentina.

Vitamin A and vitamin E stock solutions of 50 mM were prepared by dissolving the compounds in absolute ethanol. Vitamin C, vitamin B1, and vitamin B6 were dissolved in sterile Milli-Q water (Millipore, Burlington, MA, USA) to obtain 50 mM solutions. A 10 mM solution of folic acid was prepared by dissolving it in a sterile sodium bicarbonate solution (pH = 8). Additionally, RES was dissolved in absolute ethanol to obtain a 10 mM stock solution. Finally, a 10 mM stock solution of CH223191 (AHR antagonist) was prepared in dimethyl sulfoxide. Any other chemicals employed were analytical grade.

Retinyl palmitate was used in cell-based assays due to its greater chemical stability in culture conditions. For molecular modeling, all-trans-retinol was selected as the major biologically active intracellular vitamin A form relevant for evaluating potential interactions with the AHR binding pocket.

#### 4.1.2. Cells and Virus

Vero cells (African green monkey kidney epithelial cells; ATCC CCL-81), widely used for flavivirus propagation due to their high permissiveness to viral replication, and hTERT-RPE1 (hRPE1) cells derived from human telomerase reverse transcriptase-immortalized retinal pigment epithelial cells (ATCC CRL-4000) were used. Both cell lines were cultured in Eagle’s minimal essential medium (MEM; Gibco, Thermo Fisher Scientific, Waltham, MA, USA) supplemented with 5% fetal bovine serum and 50 μg/mL gentamicin (Sigma Aldrich, St. Louis, MO, USA).

ZIKV, Argentine strain INEVH116141, was provided by the Instituto Nacional de Enfermedades Virales Humanas “Dr. J. I. Maiztegui” (Pergamino, Argentina).

All experiments involving ZIKV infection were conducted under biosafety level 2 (BSL-2) conditions, in accordance with the biosafety regulations of the Environmental Health and Safety Office, Faculty of Exact and Natural Sciences, University of Buenos Aires.

#### 4.1.3. Cell Viability Assay

Vero and hRPE1 cells were seeded in 96-well plates and exposed to different concentrations of vitamins (50–800 µM) and RES (6–50 µM) individually. Among the vitamins tested, vitamin A was selected for subsequent combination studies with RES. After 72 h, cells were fixed with 10% formaldehyde and stained with 0.05% crystal violet in 10% ethanol. Then, the bound dye was solubilized with a solution containing 50% ethanol and 0.1% acetic acid solution. Absorbance was measured at 570 nm and cell viability was normalized to the absorbance of untreated control cells.

#### 4.1.4. Infections and Treatments

Stocks of vitamins were diluted in MEM supplemented with 1.5% fetal bovine serum to concentrations of 400 and 800 µM. In the case of vitamin A, concentrations ranging from 50 to 800 μM were tested. Additionally, RES (6–50 µM) and CH223191 (10 μM) solutions were prepared.

To evaluate the antiviral activity, Vero and hRPE1 cells were infected with ZIKV at a multiplicity of infection (MOI) of 0.1 for 1 h at 37 °C. Subsequently, the inocula were removed and the prepared solutions were added for 48 h at 37 °C. After this time, the supernatants were harvested and titrated by plaque assay. On the other hand, for pre-treatments, vitamin A (800 μM) and CH223191 (10 μM) solutions were added to hRPE1 cells for 2 h at 37 °C. Then, cells were infected for 1 h (MOI = 0.1). After this time, fresh medium was added and the cells were incubated for 24 h at 37 °C. Cell cultures were preserved for quantification by RT-qPCR.

#### 4.1.5. Plaque Assay

Vero cells were seeded into 24-well microplates and grown overnight. Ten-fold dilutions of the supernatants were added to cell monolayers and then incubated at 37 °C for 1 h. After incubation, the inocula were removed and monolayers were covered with 500 μL of MEM containing 1% methylcellulose. The cells were incubated at 37 °C for 72 h. Finally, plaques were fixed with 10% formaldehyde and stained with 1% crystal violet in 10% ethanol and counted. The virus titer was expressed as plaque-forming units per milliliter (PFU/mL).

#### 4.1.6. Quantitative Reverse Transcription Polymerase Chain Reaction (RT-qPCR)

Total RNA was extracted from cell cultures using TRI Reagent^®^ MRC (Molecular Research Center, Inc., Cincinnati, OH, USA) and quantified using a Nanodrop 2000 spectrophotometer (Thermo Fisher Scientific Inc., Waltham, MA, USA). Subsequently, cDNA was synthesized using the Moloney Murine Leukemia Virus Reverse Transcriptase (MMLV-RT) and random hexamer primers. Target sequences were amplified in a Bio-Rad iCycler with MyiQ2 Two-Color RT-PCR Detection System using FastStart™ (Thermo Fisher Scientific Inc., Waltham, MA, USA) Universal SYBR^®^ Green Master (Rox) (Thermo Fisher Scientific Inc., Waltham, MA, USA). Gene expression analyses were performed using three independent biological replicates. Primer specificity was confirmed by melting curve analysis, showing a single peak for each amplicon. Primer pairs were optimized under the selected reaction conditions and showed comparable amplification performance. Expression levels were normalized to the endogenous control β-actin (*ACTB*), and relative expression was calculated using the 2^−∆∆Ct^ method [44]. The primers used are listed in Table 2.

#### 4.1.7. Combination Therapy

Drug interaction was evaluated by testing serial 2-fold dilutions of vitamin A (25–800 μM) with RES (3–50 μM) in hRPE1 cells infected with ZIKV (MOI = 0.1) for 48 h at 37 °C. The viral titers (PFU/mL) were determined by the plaque assay. SynergyFinder (version 3) was employed to analyze the interactions of the phytochemicals and calculate the delta-score [45].

### 4.2. In Silico Assays

#### 4.2.1. Preparation of Protein Structure

The 3D structure of the PAS-B domain (PDB ID: 7ZUB) [46] was downloaded from the RCSB Protein Data Bank [47] (https://www.rcsb.org/, accessed on 4 May 2022). Residues 285–388 were selected and used for docking and molecular dynamics simulations. Gasteiger charges were incorporated into the protein and atoms were assigned to AD4 using Autodock tools 1.5.6. In addition to this, polar hydrogens were added.

#### 4.2.2. Ligands Preparation

The 3D ligand structures were drawn in Avogadro 1.93.0 [48] and optimized at the CAM-B3LYP/6-31G(d) level of theory using Gaussian 16 [49]. Afterwards, the pdb files were generated by Molden [50] and the pdbqt files were created by Autodock tools 1.5.6 [51].

#### 4.2.3. Molecular Docking

AutoDock Vina v1.2.5 was used to perform molecular docking calculations [52]. Docking simulations were conducted using a focused docking strategy targeting the PAS-B ligand-binding pocket, defined by a grid box centered at x = 159.99, y = 161.352, and z = 163.234, with dimensions of 25.36 × 25.36 × 25.36 Å^3^. As a first check, the protocol was validated by re-docking for reproducing the experimental pose of the co-crystallized ligand, indirubin.

To ensure extensive conformational sampling and assess the robustness of the predicted binding modes, multiple independent docking runs were performed using increasing exhaustiveness values ranging from 8 to 64. For each exhaustiveness setting, at least two independent simulations were carried out, and the resulting binding poses were ranked according to their predicted binding affinities. The most favorable binding modes were selected based on both predicted affinity and consistency across independent runs.

Docked complexes were visually inspected and analyzed using UCSF Chimera version 1.17.3 [53].

#### 4.2.4. Molecular Dynamics

The most stable docked complex for each ligand was used as starting geometry for the MD simulations.

The protein–ligand complexes were constructed using the AMBER22 *tleap* module, while ligand parameters were generated with *antechamber* and *parmchk2* for the parametrization of the non-protein components. Partial atomic charges were assigned using the semiempirical AM1 quantum model, as implemented within the sqm module of AMBER [54]. The complex was solvated within a box of TIP3P water molecules, with 10 Å offset and as many Na+ and Cl- counterions as required for electroneutrality. The system was first minimized with strong harmonic constraints for the protein/ligand complex (250 steps) followed by 6500 steps for the whole system. The system was then gradually heated up to 300 K over 100 ps in the NVT ensemble, applying positional restraints to the protein backbone and using the Andersen thermostat [55]. Finally, production simulations of 100 ns were carried out in the NPT ensemble at 300 K and 1 atm.

Electrostatic interactions were computed using the Particle Mesh Ewald (PME) method with a cutoff of 10 Å [56]. Bonds involving hydrogen atoms were constrained using the SHAKE algorithm, allowing for an integration time step of 0.002 ps. Simulations were performed using the pmemd.cuda module of the AMBER22 package [57] employing the GAFF2 force field for the ligands and ff18SB for the protein [58]. Trajectories were analyzed using the cpptraj module of AMBER. Binding free energy calculations were carried out using the Molecular Mechanics–Poisson–Boltzmann Surface Area (MM-PBSA) method as implemented in the MMPBSA module of AMBER18, applying both Poisson–Boltzmann (PB) and Generalized Born (GB) implicit solvent models [59]. System stability was assessed by monitoring backbone RMSD along the trajectories (see for example Figure S1). Free energies of binding estimates were calculated over the last 10 ns of each simulation, after confirming structural convergence. Clustering analyses and visualization of representative conformations were performed using Chimera 1.17.3 [53], and molecular graphics were generated using Chimera and VMD 1.9.5 [60].

### 4.3. Statistical Analysis

Differences between experimental groups were analyzed by one-way analysis of variance (ANOVA) following Dunnett’s or Tukey’s post hoc tests using GraphPad Prism software 8.0.1 (San Diego, CA, USA). Dunnett’s test was used to compare each experimental group with the control, while Tukey’s test was used for comparisons among experimental groups. Statistical significance was set at *p* < 0.05.

## 5. Conclusions

Overall, our findings demonstrate that vitamin A restricts ZIKV replication through modulation of host antiviral pathways, potentially involving regulation of AHR-associated signaling and enhancement of IFN-I responses. The enhanced inhibition observed in combination with RES highlights the potential of simultaneously targeting complementary host pathways to improve antiviral efficacy. In this context, the combination of vitamin A and RES emerges as a promising host-directed strategy, supporting the repurposing of well-characterized bioactive compounds for the development of novel therapeutic approaches against ZIKV. More broadly, these results underscore the potential of exploiting host-modulatory bioactives as complementary tools to combat emerging viral infections for which specific antiviral therapies remain limited.

## Figures and Tables

**Table 1:** MMGBSA binding free energy calculations for ligands docked to the AHR PAS-B domain.

Ligand	Structure	MMGBSA–GB (Kcal/mol)
Indirubin (re-docking)	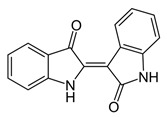	−36.55
CH223191	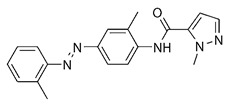	−42.76
TCDD	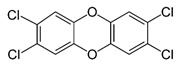	−40.11
All-trans-retinol	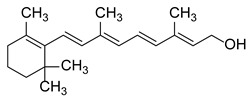	−40.85
Vitamin B6 (pyridoxine)	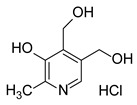	−20.13

**Table 2:** Primers used in RT-qPCR assays.

Primer	*Forward* (5′-3′)	*Reverse* (5′-3′)
*ACTB*	TTAGTTGCGTTACACCCTTTCTTG	TCACCTTCACCGTTCCAGTTT
*NS5*-ZIKV	GCCGCCACCAAGATGAACTGATTG	GCAGTCTCCCGGATGCTCCATC
*AHR*	ATCCTTCCAAGCGGCATAGAGACC	CAAAGAAGCTCTTGGCTCTCAGG
*TDO*	GAGGAACAGGTGGCTGAATTT	GCTCCCTGAAGTGCTCTGTA
*CYP1A1*	GAACTGCTTAGCCTAGTCAACCTG	AGAATAGGGATGAAGTCAGCTGGG
*IFNB1*	TAGCACTGGCTGGAATGAGA	TCCTTGGCCTTCAGGTAATG

## Data Availability

The original contributions presented in this study are included in the article. Further inquiries can be directed to the corresponding authors.

## Supplementary Materials

The following supporting information can be downloaded at: https://www.mdpi.com/article/10.3390/pathogens15050518/s1, Figure S1: RMSD of the AHR complexes with CH223191 and vitamin A obtained from MD simulations; Figure S2: Redocking validation; Figure S3: Representative clusters from AHR–vitamin A MD simulations Table S1: Two-dimensional chemical structures of vitamins.
