# Supplementary material for: Vitamin A Modulates AHR Signaling and Restricts Zika Virus Replication in Human Retinal Pigment Epithelial Cells: Insights from Molecular Modeling and Antiviral Assays
Source: Pathogens. 2026 May 12;15(5):518. doi: 10.3390/pathogens15050518 (PMC13209694; doi:10.3390/pathogens15050518)
Supplement: Supplementary file 1 [file pathogens-15-00518-s001.zip › pathogens-4274574-supplementary.pdf]

Vitamin A Modulates AHR Signaling and Restricts Zika Virus Replication in Human Retinal Pigment Epithelial Cells: Insights from Molecular Modeling and Antiviral Assays

Agostina B. Marquez [1,2,†], Priscila A. Lanza Castronuovo [3,4,†], Cecilia L. Barbieri [3], Mayra A. Castañeda Cataña [1,2], Claudia S. Sepúlveda [1,2], Agustina Alaimo [2], D. Mariano A. Vera [3,*] and Cybele C. García [1,2,*]

[1] Laboratorio de Estrategias Antivirales, Departamento de Química Biológica, Facultad de Ciencias Exactas y Naturales, Universidad de Buenos Aires (UBA), Intendente Güiraldes 2160, Ciudad Autónoma de Buenos Aires 1428, Argentina
[2] Instituto de Química Biológica de la Facultad de Ciencias Exactas y Naturales (IQUIBICEN), Consejo Nacional de Investigaciones Científicas y Técnicas (CONICET)–UBA, Intendente Güiraldes 2160, Ciudad Autónoma de Buenos Aires 1428, Argentina
[3] QUIAMM-INBIOTEC, Departamento de Química y Bioquímica, Facultad de Ciencias Exactas y Naturales, Universidad Nacional de Mar del Plata, Funes 3350, Mar del Plata 7600, Argentina
[4] Comisión de Investigaciones Científicas (CIC), Calle 526 entre 10 y 11, La Plata 1900, Argentina
* Correspondence: dvera@mdp.edu.ar (D.M.A.V.); cygarcia@qb.fcen.uba.ar (C.C.G.)
† These authors have contributed equally to this work.

Supporting Information

Table S1. Two-dimensional chemical structures of vitamins.

Vitamins	Chemical name	Structures
Vitamin B1	Thiamine hydrochloride	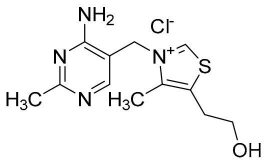
Vitamin B9	Folic acid	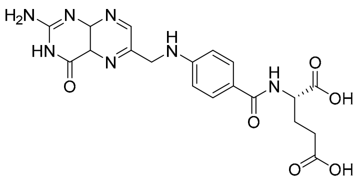
Vitamin C	Ascorbic acid	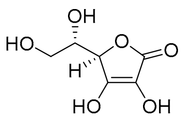
Vitamin E	A-tocopherol	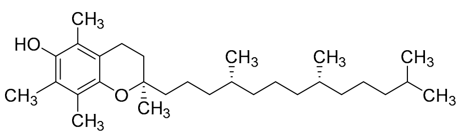

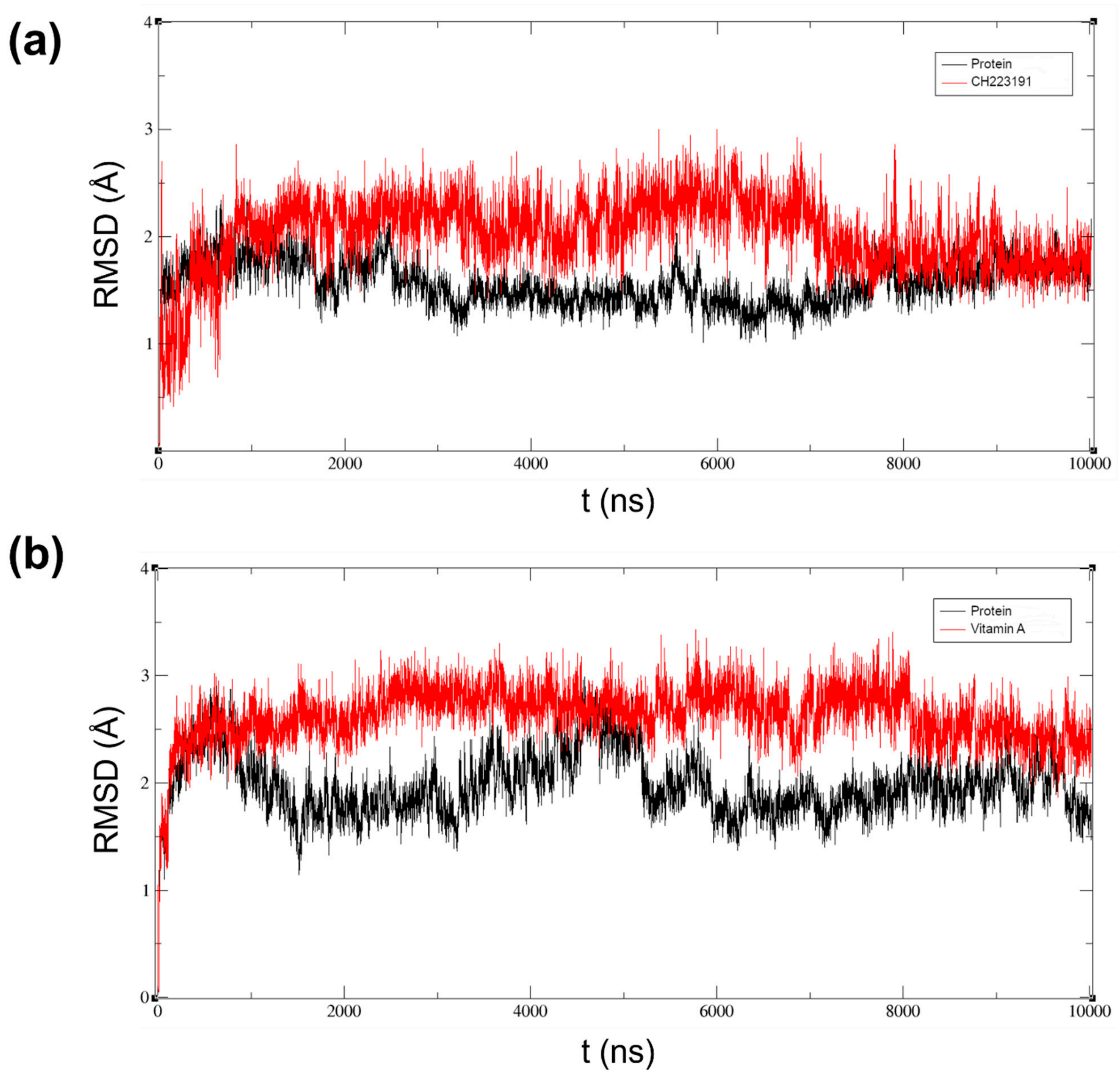

Figure S1. RMSD of the AHR complexes with **(a)** CH223191 and **(b)** Vitamin A obtained from molecular dynamics simulations. The RMSD values for the ligand (red line) and the protein (black line) are shown as a function of time.

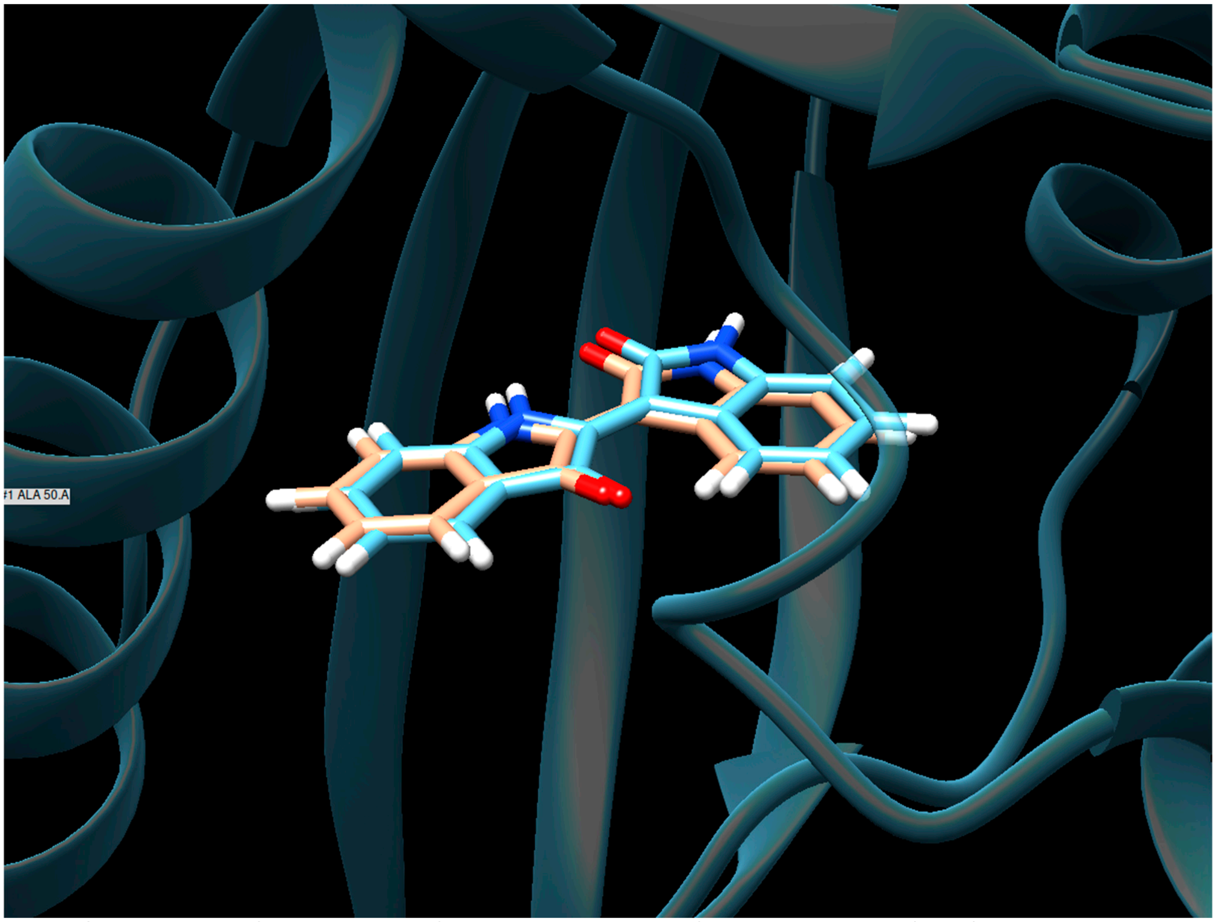

Figure S2. Redocking. Superimposition of the experimental pose of the ligand indirubin (beige) and the lowest energy pose obtained by Autodock Vina (cyan).

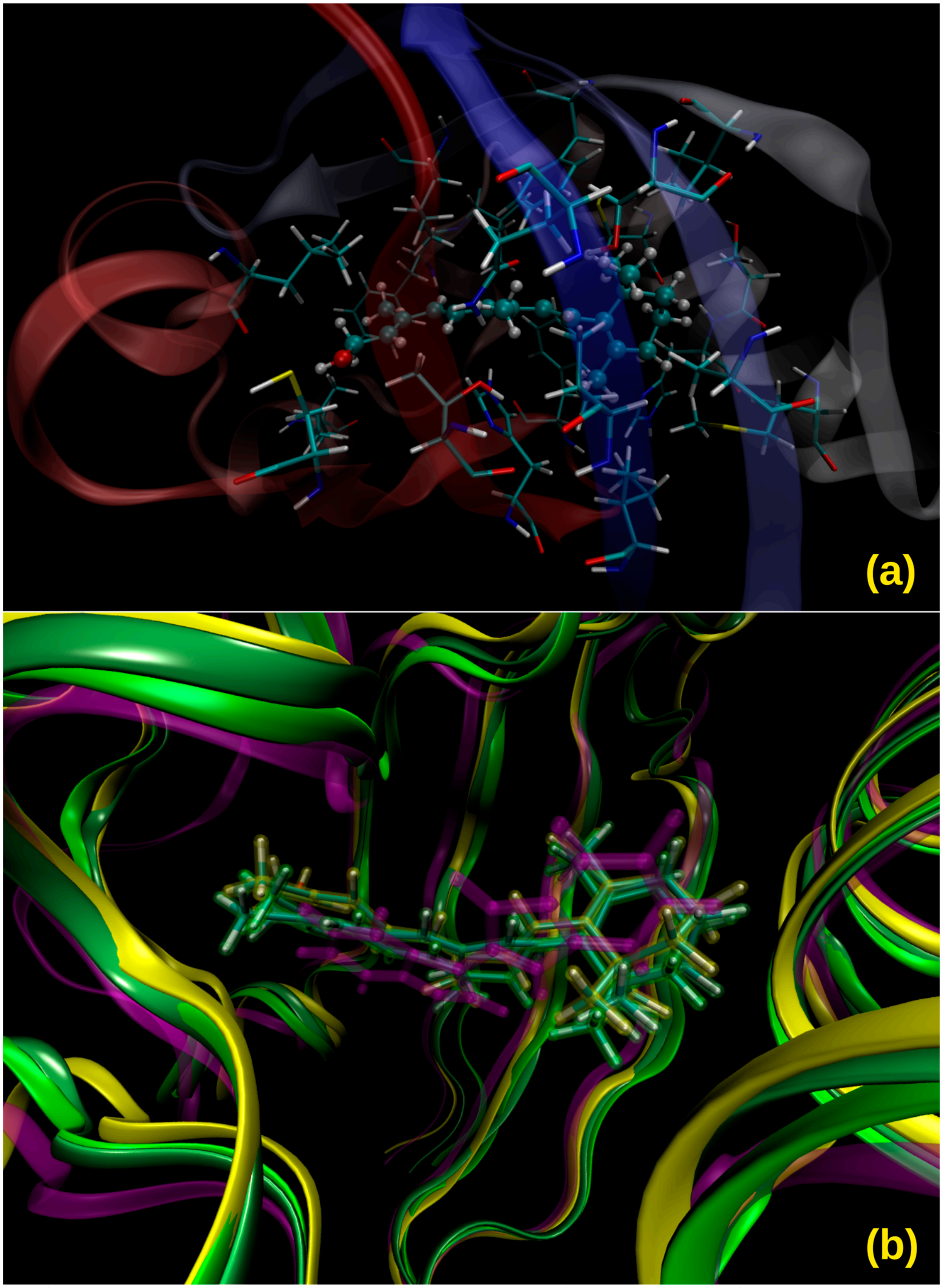

Figure S3. **(a)** Representative snapshot of the most populated cluster from the simulation of the complex with vitamin A (last 90 ns analysis), cartoon colored according to the sequence (from red to blue), **(b)** Representative snapshots (yellow-green) of the first three most populated clusters of the last 90 ns of simulations of the complex with vitamin A superimposed to the complex with the crystallographic reference indirubin (translucent violet).
